# The addition of celecoxib improves the antitumor effect of cetuximab in colorectal cancer: role of EGFR-RAS-FOXM1-β-catenin signaling axis

**DOI:** 10.18632/oncotarget.15567

**Published:** 2017-02-21

**Authors:** Araceli Valverde, Jon Peñarando, Amanda Cañas, Laura M. López-Sánchez, Francisco Conde, Silvia Guil-Luna, Vanessa Hernández, Carlos Villar, Cristina Morales-Estévez, Juan de la Haba-Rodríguez, Enrique Arand o, Antonio Rodríguez-Ariza

**Affiliations:** ^1^ Instituto Maimónides de Investigación Biomédica de Córdoba (IMIBIC), Córdoba, Spain; ^2^ Centro de Investigacion Biomédica en Red de Cáncer, Madrid, Spain; ^3^ Unidad de Gestión Clínica de Anatomía Patológica, Hospital Universitario Reina Sofía, Córdoba, Spain; ^4^ Unidad de Gestión Clínica de Oncología, Hospital Universitario Reina Sofía, Córdoba, Spain

**Keywords:** β-catenin, colorectal cancer, COX-2, EGFR, FOXM1

## Abstract

Here we showed that the addition of the COX-2 inhibitor celecoxib improved the antitumor efficacy in colorectal cancer (CRC) of the monoclonal anti-EGFR antibody cetuximab. The addition of celecoxib augmented the efficacy of cetuximab to inhibit cell proliferation and to induce apoptosis in CRC cells. Moreover, the combination of celecoxib and cetuximab was more effective than either treatment alone in reducing the tumor volume in a mouse xenograft model. The combined treatment enhanced the inhibition of EGFR signaling and altered the subcellular distribution of β-catenin. Moreover, knockdown of FOXM1 showed that this transcription factor participates in this enhanced antitumoral response. Besides, the combined treatment decreased β-catenin/FOXM1 interaction and reduced the cancer stem cell subpopulation in CRC cells, as indicated their diminished capacity to form colonospheres. Notably, the inmunodetection of FOXM1 in the nuclei of tumor cells in human colorectal adenocarcinomas was significantly associated with response of patients to cetuximab. In summary, our study shows that the addition of celecoxib enhances the antitumor efficacy of cetuximab in CRC due to impairment of EGFR-RAS-FOXM1-β-catenin signaling axis. Results also support that FOXM1 could be a predictive marker of response of mCRC patients to cetuximab therapy.

## INTRODUCTION

Cetuximab is a human-murine chimeric monoclonal antibody that was approved by the FDA in 2004 for the treatment of metastatic colorectal cancer (mCRC), as a single agent or in combination with chemotherapy [1, 2]. Cetuximab competes with epidermal growth factor (EGF) for binding to the extracellular domain of EGF receptor (EGFR), thereby inhibiting EGF-induced tyrosine kinase phosphorylation, cell growth and resistance to apoptosis [3]. Cetuximab inhibits the proliferation of tumor cells expressing EGFR and increases the cytotoxic activity of chemotherapy and radiotherapy [2, 4]. However, due to refractory or resistant disease, only a fraction of patients obtain clinical benefit from treatment with this monoclonal antibody [5, 6]. The concomitant inhibition of EGFR and other signaling pathways has been proven effective in colorectal cancer cells [7, 8]. Therefore, the use of cetuximab in combination with other drugs may constitute an alternative strategy to increase its therapeutic effect. The selective cyclooxygenase-2 (COX-2) inhibitor celecoxib is useful in preventing polyp formation in familial adenomatous polyposis (FAP) patients, a population at high risk for colorectal cancer development [9]. However, studies show that inhibition of COX-2 does not appear to add benefit when combined with chemotherapy in mCRC [10, 11].

The COX-2 and EGFR pathways mutually enhance their pro-tumorigenic effects in different tumor types [12] and we have previously shown that the combined treatment of the anti-EGFR drug AEE788 and the specific COX-2 inhibitor celecoxib demonstrated enhanced anti-tumoral efficacy in CRC cells [13]. Therefore, the combination of cetuximab with celecoxib could also improve the antitumor efficacy of this monoclonal antibody, and increase therapeutic benefit in mCRC. In the present study we show that celecoxib enhances the antitumor efficacy of cetuximab in CRC and reduces colon cancer stem cells tumor subpopulation, supporting that the combination of EGFR and COX-2 inhibitors could improve current mCRC therapies. Our results also highlight the importance of the EGFR-Ras-FOXM1-β-catenin signaling axis in CRC and indicate that the expression of the transcription factor FOXM1 in colorectal tumors may be a potential predictive marker of response to cetuximab therapy in this disease.

## RESULTS

### Cetuximab/celecoxib combined treatment inhibits cell proliferation and induces apoptosis in colorectal cancer cells

**Figure 1:**
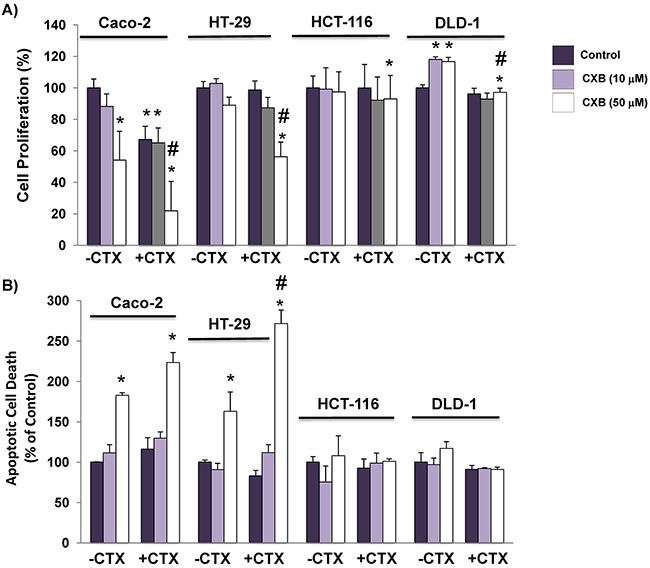
The addition of celecoxib enhances the capacity of cetuximab to inhibit cell proliferation and to induce apoptosis in colorectal cancer cells **A**. Cell proliferation and, **B**. The fraction of apoptotic cells was evaluated after 72h of treatment with cetuximab (CTX, 100 μg/ml) alone or in combination with different doses (10 μM and 50 μM) of celecoxib (CXB). Data are means ± SEM of three independent experiments (*p <0.05, compared with the control; # p<0.05, compared with cetuximab-treated cells).

First, we analyzed the anti-proliferative and anti-apoptotic activity of combined cetuximab (100 mg / ml)/celecoxib (10 μM and 50 μM) treatment in CRC cells (Figure 1A). In cell proliferation assays, both cetuximab and celecoxib showed antiproliferative capacity in Caco-2 cells when administered separately, but a more potent inhibitory effect was obtained when both treatments were combined, reducing cell proliferation by 80%. Furthermore, only the combination of both drugs could reduce proliferation by 50% in cetuximab-resistant HT-29 cells. On the other hand, proliferation of KRAS-mutated HCT116 or DLD-1 cells were unaffected by those treatments. Cell cycle analyses showed that the anti-proliferative effect observed in Caco-2 and HT-29 cells was associated with G1 cell cycle arrest (Supplementary Figure 1). The analysis of the capacity of cetuximab and/or celecoxib to induce apoptosis (Figure 1B) confirmed that only the combined treatment exerted a strong apoptotic effect on Caco-2 and HT-29, but not in HCT-116 or DLD-1 cells.

### The combination of cetuximab with celecoxib improves the inhibition the EGFR-directed signaling pathway in colorectal cancer cells

**Figure 2:**
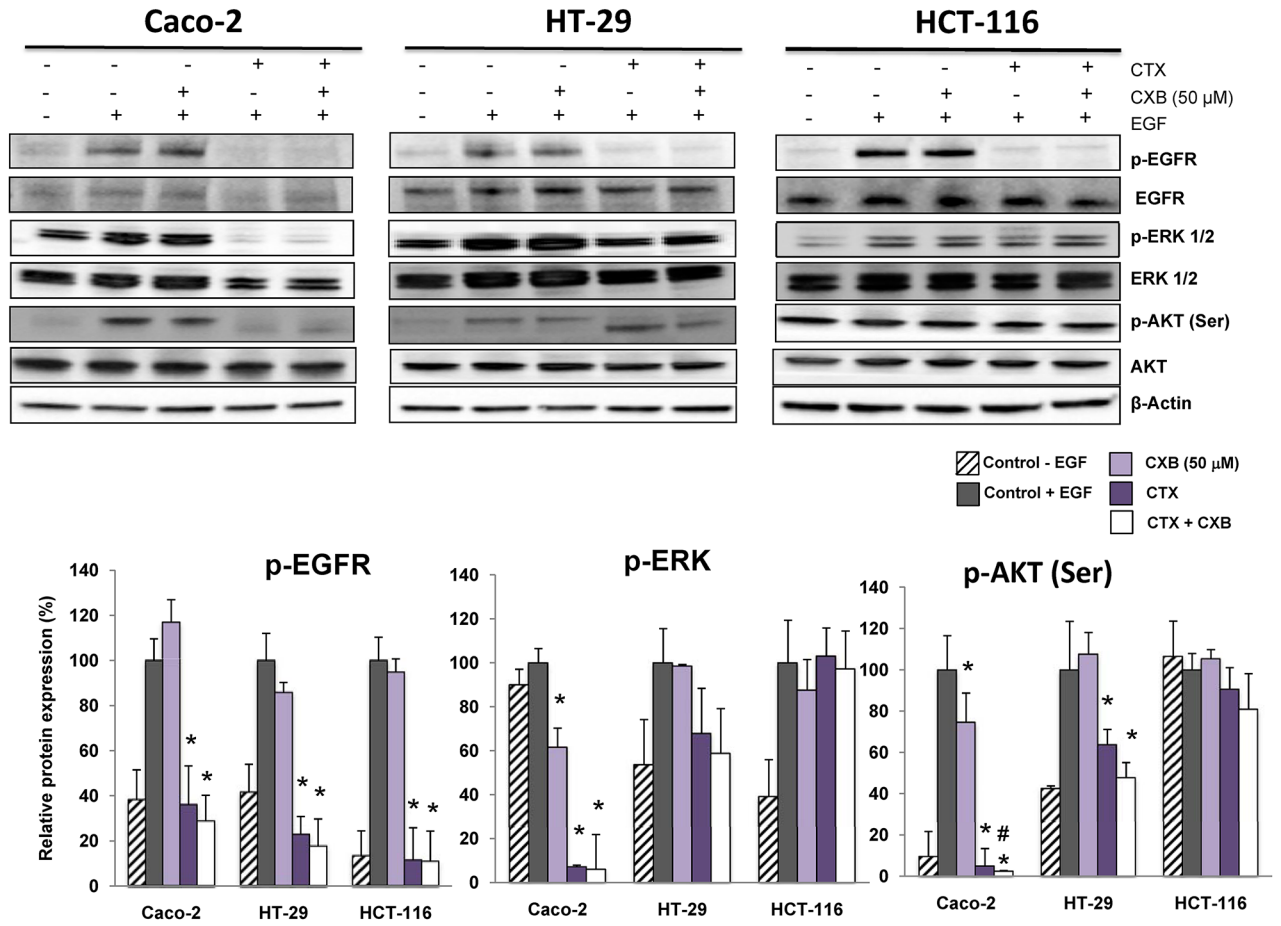
The combination of cetuximab with celecoxib improves the inhibition the EGFR-directed signaling pathway in colorectal cancer cells The phosphorylated and non-phosphorylated forms of EGFR, ERK 1/2 and AKT(Ser) were detected by Western-blot using specific antibodies. Cells were treated for 1h with cetuximab (CTX, 100 μg/ml) alone or in combination with celecoxib (CXB, 50 μM) and then treated with EGF (100 ng/mL) for 5 min. The expression level of β-actin was included as loading control. The corresponding densitometric analysis is also shown. Data are means ± SEM of three independent experiments (*p <0.05, compared with the control; # p<0.05, compared with cetuximab-treated cells).

Phosphorylation of EGFR, ERK and AKT was next analyzed to evaluate the effects of cetuximab and/or celecoxib on the EGFR signaling pathway. As shown in Figure 2, cetuximab effectively inhibited EGFR phosphorylation in Caco-2, HT-29 and HCT-116 cells. However, as indicated the inhibition of EGF-induced phosphorylation of ERK1/2 and Akt Ser, the combination of cetuximab with celecoxib inhibited EGFR signaling in Caco-2 and HT-29 cells, but not in KRAS-mutated HCT-116 cells. Therefore, the antiproliferative activity of the combined cetuximab/celecoxib treatment in colon cancer cells is strongly dependent on the mutational status of KRAS. Indeed, Caco-2 and HT-29 cells transfected with mutated KRAS were resistant to the anti-proliferative effect of cetuximab and celecoxib, alone or in combination (Supplementary Figure 2). Likewise, transfection of mutated KRAS into Caco-2 cells abrogated the apoptotic effect of the cetuximab/celecoxib combined treatment (121±11 control vs 64 ± 8 in mutated KRAS-transfected cells, percentage of untreated control cells; p< 0.05). In agreement with cell proliferation and cell death data, the addition of celecoxib to cetuximab enhanced the inhibition of ERK 1/2 and AKT phosphorylation, especially in Caco-2 cells, (Figure 2).

### The combination of cetuximab with celecoxib improves the anti-tumor effect in a xenograft tumor model

**Figure 3:**
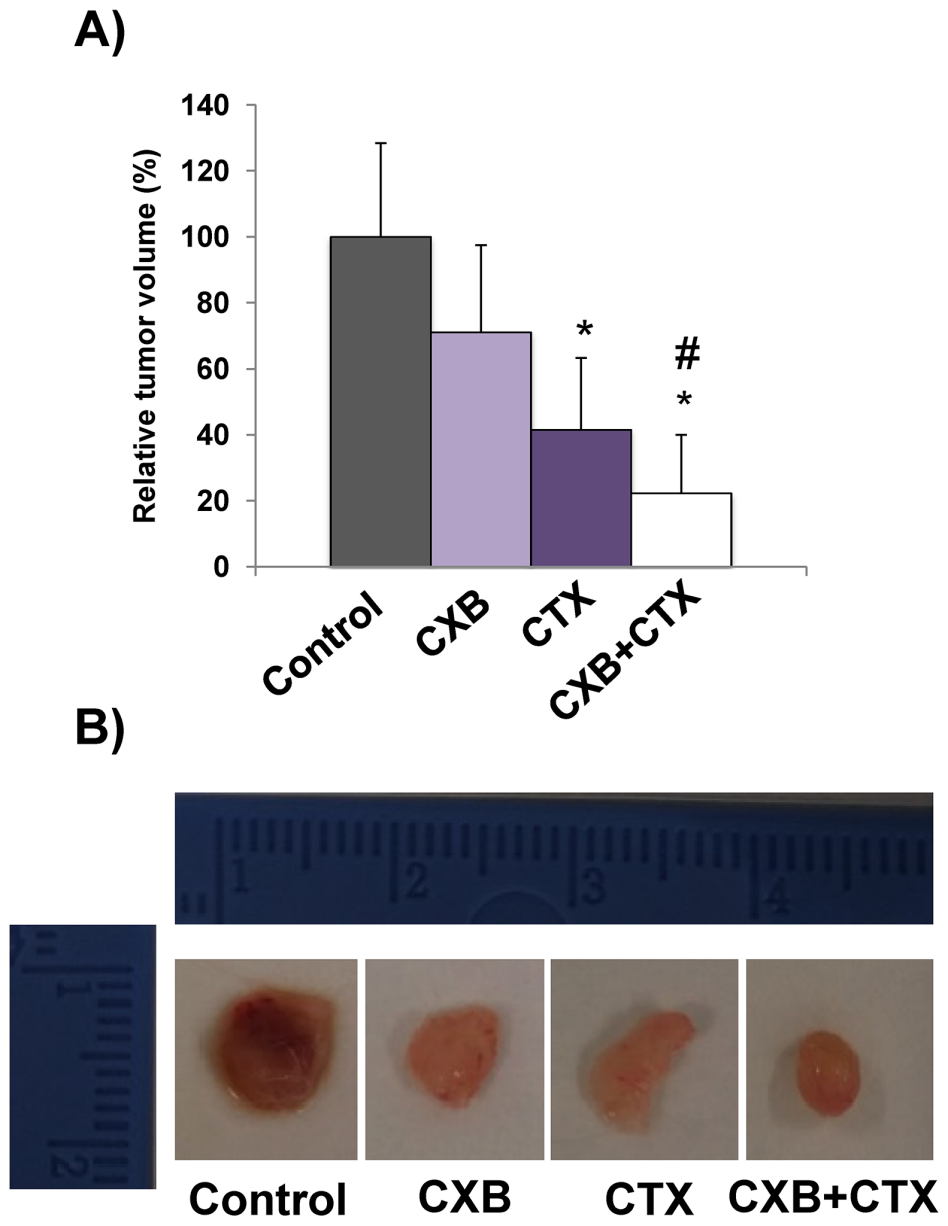
The combination of cetuximab with celecoxib improves the antitumor activity against Caco-2 tumor xenografts Male nude mice (NOD-SCID) were xenografted with Caco-2 colorectal carcinoma cells. When tumors reached approximately 100–150 mm^3^ volume, cetuximab (CTX, 20 mg/kg) and/or celecoxib (CXB, 60 mg/kg) were administered twice a week for 24 days. **A**. Relative tumor volume in the different treatment groups (*p <0.05, compared with the control; # p<0.05, compared with cetuximab-treated cells). **B**. Representative images of excised tumors.

To confirm the *in vitro* results, the antitumor effect of cetuximab and celecoxib, alone or in combination, was then evaluated in a xenograft model. To this end, Caco-2 cells were grafted into immunocompromised mice and once the tumors were generated, the animals were treated with each drug separately or in combination. As shown in Figure 3, the combination of cetuximab and celecoxib exerted a better antitumor effect than either drug alone, with a significantly higher reduction in tumor volume.

### The combined treatment of cetuximab and celecoxib alters the subcellular distribution of β-catenin in Caco-2 cells

**Figure 4:**
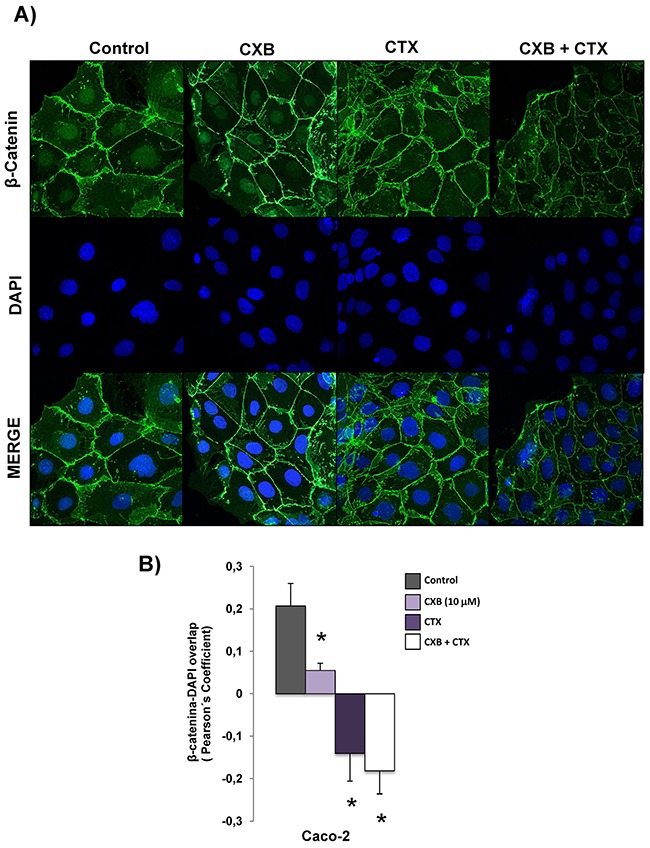
Combined Celecoxib/Cetuximab treatment alters subcellular distribution of β-catenin in Caco-2 cells **A**. To determine subcellular localization of β-catenin in Caco-2, cells were exposed for 6 h to Celecoxib (CXB, 10 μM) alone or in combination with Cetuximab (CTX, 100 μg/ml) and the presence of EGF (100 ng/ml) and then, cells were stained for β-catenin immunofluorescence (green) and counterstained with DAPI (blue). Merged images of β-catenin and DAPI staining are also shown. Final magnification: X400. **B**. Pearson's coefficient analysis was performed for the co-localization in nuclei cell of β-catenin and DAPI. Data are means ± SEM of three independent experiments (*p <0.05, compared with the control).

We have previously reported that the combination of the anti-EGFR tyrosine kinase inhibitor AEE788 with celecoxib altered the subcellular distribution of β-catenin in CRC cells, Therefore, we next examined whether treatment with cetuximab and/or celecoxib could exert a similar effect on Caco-2 cells. As shown in Figure 4, confocal microscopy analyses revealed that in untreated cells, the expression of β-catenin was detected in membrane, cytoplasm and nucleus. However, treatment with cetuximab and, especially the combined treatment cetuximab/celecoxib drastically reduced nuclear β-catenin levels in Caco-2 cells.

### FOXM1 participates in the response of colorectal cancer cells to treatment with cetuximab and/or celecoxib

**Figure 5:**
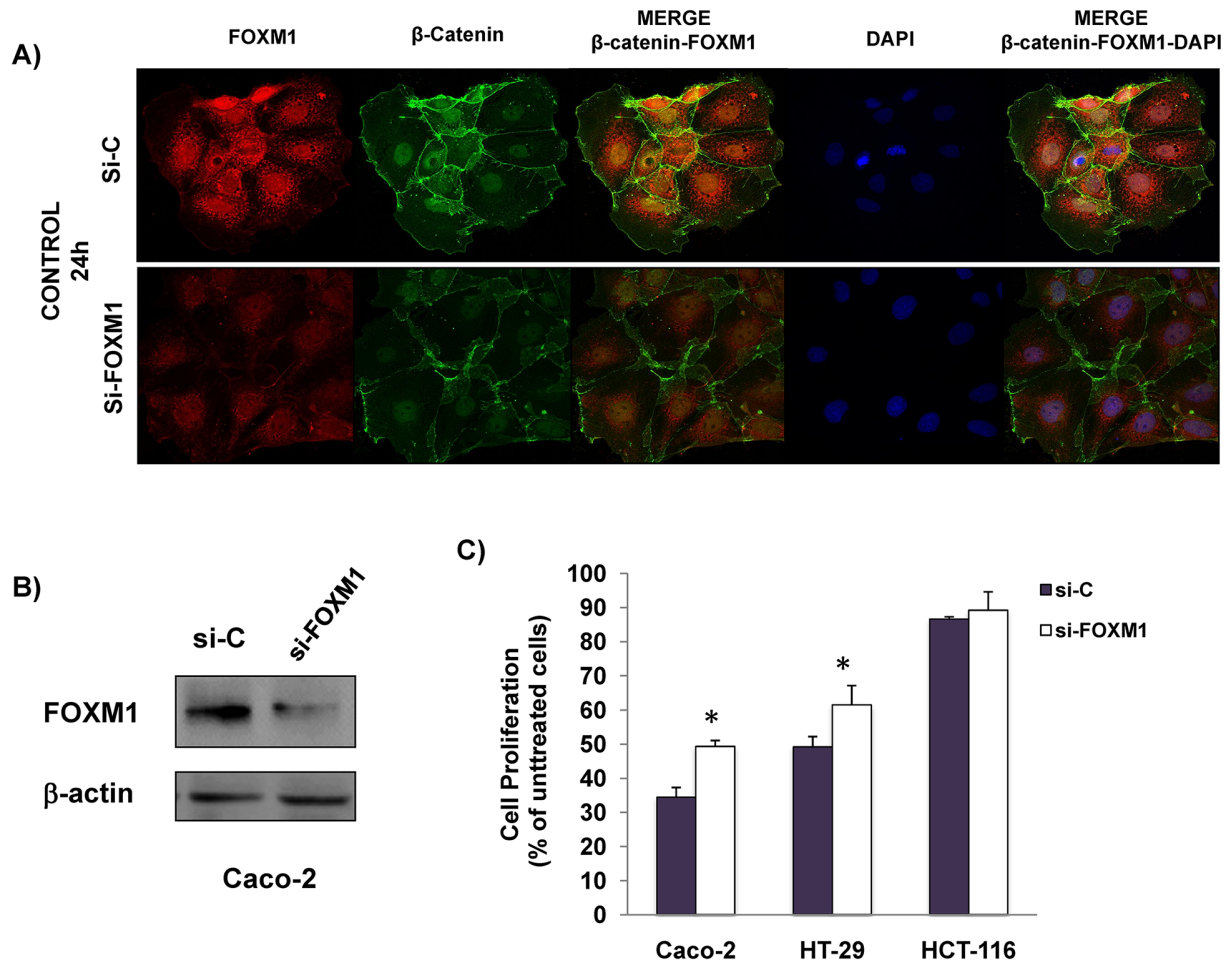
The anti-proliferative effect is reduced by specific FOXM1-silencing in CRC cells Cells were transiently transfected with a siRNA control (si-C) and a specific siRNA FOXM1 (si-FOXM1) for 24 h and was analyzed: **A**. FOXM1 and β-catenin expression in untreated Caco-2 cells by inmmunofluorescence. Merged images of β-catenin and DAPI staining are also shown. Final magnification: X400 and, **B**. FOXM1 expression was analized by immunoblotting in whole cell lysates. The expression level of β-actin was included as loading control. **C**. Cells were transiently transfected with siRNA control (si-C) and a specific si-FOXM1 for 24 hours and then treated with celecoxib (50 μM) and cetuximab (100 μg/ml). The cell proliferation inhibition was determined after 72 hours of treatment using the incuCyte ZOOM® Software 2015A. Data are means ± SEM of three independent experiments (*p <0.05, compared with si-C-transfected cells).

We and others have previously shown that FOXM1 may play an important role in the response of colorectal cancer cells to anti-EGFR treatment [13, 14]. Therefore, we next explored whether FOXM1 participates in the anti-proliferative effect of cetuximab and/or celecoxib. To this end, the expression of this transcription factor was specifically silenced in Caco-2 cells. Confocal microscopy (Figure 5A) and western blot analysis (Figure 5B) showed that specific silencing of FOXM1 in Caco-2 cells caused a significant reduction in the expression of FOXM1 protein. Besides, the specific knockdown of this transcription factor also significantly reduced the nuclear β-catenin levels. Importantly, the specific silencing of FOXM1, significantly reduced the antiproliferative effect of cetuximab/celecoxib treatment in Caco-2 and HT-29 cells (Figure 5C).

### The combined treatment of cetuximab and celecoxib alters the interaction of β-catenin with FOXM1-in colorectal cancer cells

**Figure 6:**
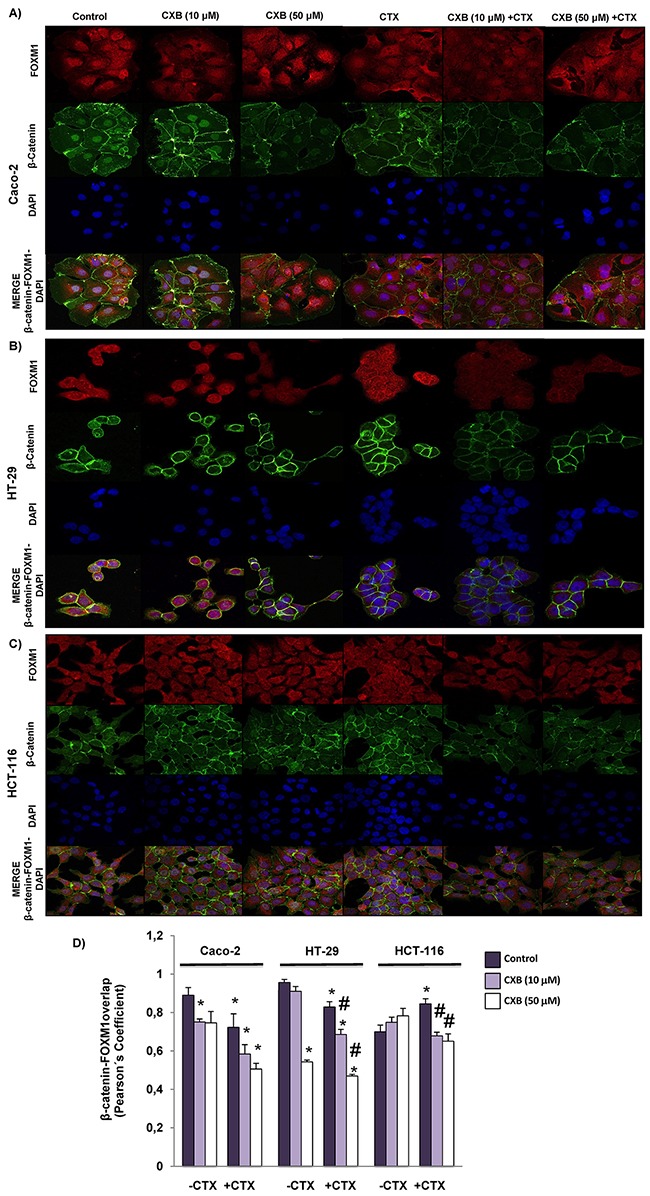
Combined Celecoxib/Cetuximab treatment impairs FOXM1-β-catenin interaction in colorectal cancer cells To determine subcellular localization of β-catenin and FOXM1, in **A**. Caco-2, **B**. HT-29 and **C**. HCT-116, cells were exposed for 6 h to celecoxib (CXB, 10 μM or 50 μM) alone or in combination with cetuximab (CTX, 100 μg/ml) and then, cells were stained for β-catenin (green) and FOXM1 (red) immunofluorescence, and counterstained with DAPI (blue). Merged images for β-catenin, FOXM1 and DAPI staining are also shown. Final magnification: X400 (Caco-2), X600 (HCT-116 and HT-29) **D**. Pearson's coefficient analysis was performed for the co-localization in cell nuclei of β-catenin and FOXM1. Data are means ± SEM of three independent experiments (*p <0.05, compared with the control; # p<0.05, compared with cetuximab-treated cells).

**Figure 7:**
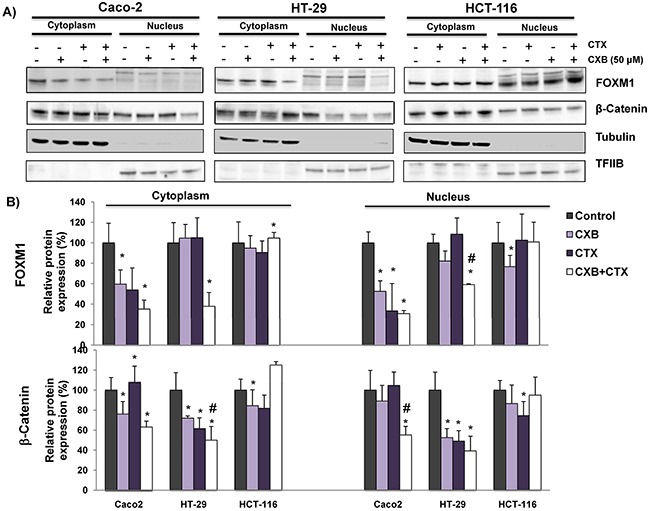
The combined Celecoxib/Cetuximab treatment alters the nuclear levels of β-catenin and FOXM1 in CRC cells To determine if FOXM1 promotes the nuclear accumulation of β-Catenin in **A**. Caco-2, HT-29 and HCT-116, cells were exposed to the indicated treatments (cetuximab, CTX; celecoxib, CXB) for 6 hours and then cytosolic and nuclear fractions were obtained. The expression of FOXM1 and β-catenin was analyzed by immunoblotting and the immunodetection of tubulin and TFIIB were included as loading controls for cytosolic and nuclear fractions, respectively. **B**. The corresponding densitometric analyses are also shown. Data are means ± SEM of three independent experiments (*p <0.05, compared with the control; # p<0.05, compared with cetuximab-treated cells).

We next decided to investigate whether the combined treatment of cetuximab and celecoxib alters the interaction of β-catenin-FOXM1 in colorectal cancer cells. As shown in Figure 6, the nuclear co-localization of β-catenin and FOXM1 was significantly lower when the Caco-2 and HT-29 cells were treated with cetuximab alone or in combination with celecoxib (Figure 6A–6B and 6D). However, neither cetuximab or celecoxib, alone or in combination, significantly altered nuclear localization of β-catenin and FOXM1 in HCT-116 cells (Figure 6C). To confirm the above results, colorectal cancer cells were exposed to the different treatments and FOXM1 and β-catenin expression in cytosolic and nuclear fractions was analyzed by western blot (Figure 7). Cetuximab and/or celecoxib largely decreased nuclear levels of β-catenin and FOXM1 in Caco-2 and HT-29 cells. In contrast, neither cetuximab, or celecoxib, alone or in combination, significantly altered nuclear levels of both proteins in HCT-116 cells.

### The combined cetuximab and celecoxib treatment decreases the capacity of colorectal cancer cells to form colonospheres

**Figure 8:**
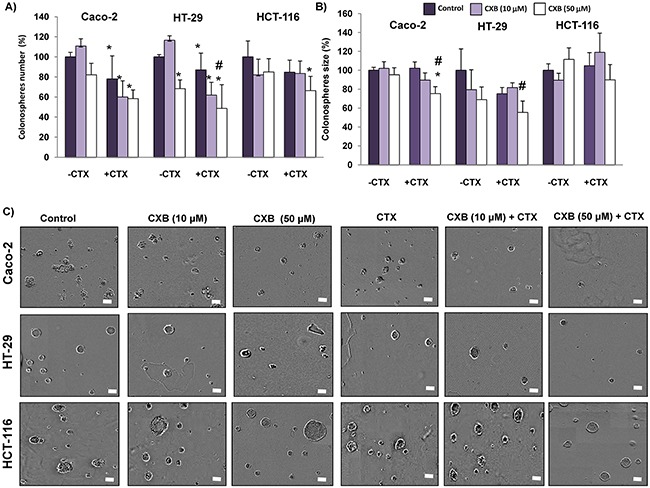
Combined Celecoxib/Cetuximab treatment in colon cancer cells impairs colonosphere formation capability Colon cancer cells were pre-treated with 10 μM or 50 μM Celecoxib (CXB) as a single agent or in combination with 100 μg/ml Cetuximab (CTX) for 48h, and then cells were seeded at clonal density with serum free medium in low-adherence plates. After seven days, **A**. the number, **B**. size and, **C**. appearance of formed colonospheres were evaluated by light microscopy. Spheres number and size were quantified with the incuCyte ZOOM® Software 2015A. (Final magnification: X40, scale bar corresponds to 100 microns). Data are means ± SEM of three independent experiments (*p <0.05, compared with the control; # p<0.05, compared with cetuximab-treated cells).

Numerous studies indicate that Wnt-β-catenin signaling contributes to tumor progression through the maintenance of a highly tumorigenic subpopulations of cancer stem cells (CSCs) [15, 16]. The formation of tumorospheres (colonospheres) *in vitro* by self-renewal is a functional assay of CSCs subpopulations in cancer cells. Therefore, we next investigated whether cetuximab and/or celecoxib treatment would impact in the capacity of colorectal cancer cells to form colonospheres. As shown in Figure 8, the pre-treatment of Caco-2 and HT-29 cells with cetuximab, alone or in combination with celecoxib reduced its ability to form colonospheres. Significantly, in the case of HCT-116, the combined treatment also significantly reduced the formation of colonospheres. Moreover, in all three cell lines the combination treatment caused the formation smaller colonospheres compared with those derived from untreated cells (Figure 8B-8C). Therefore, combination therapy notably contributed to the decrease of the subpopulation of CSCs in the three cell lines.

### The expression of the transcription factor FOXM1 in human colorectal adenocarcinomas is significantly associated with response to treatment with cetuximab

The results so far obtained indicate that the anti-proliferative response to treatment with cetuximab and/or celecoxib is related to FOXM1 and b-catenin expression and their nuclear localization in colorectal cancer cells. Therefore, we decided to analyze whether expression of FOXM1 and/or b-catenin in human colorectal tumors may constitute a predictive factor of response to anti-EGFR therapy.

**Figure 9:**
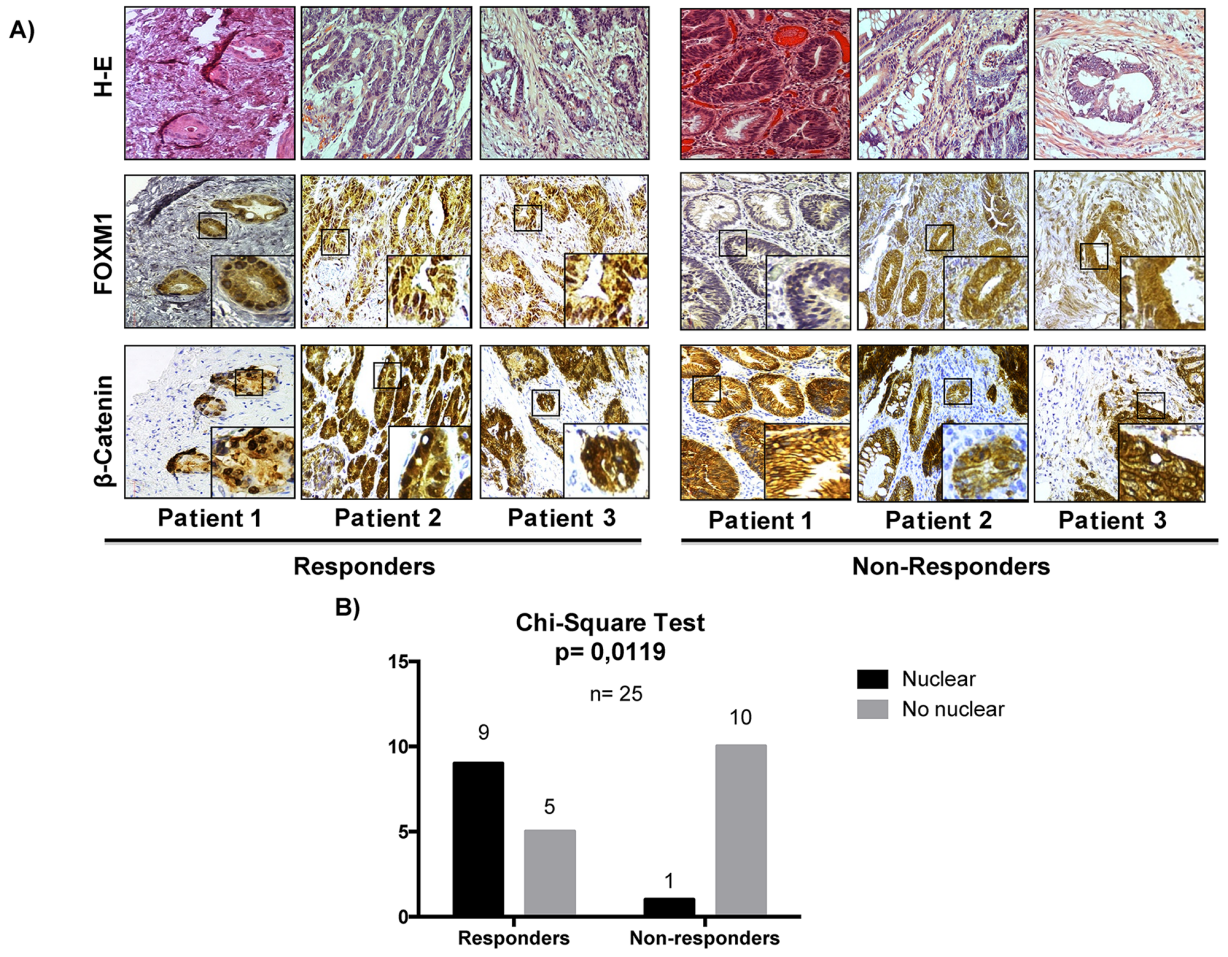
The nuclear expression of FOXM1 in colon cancer is significantly associated with a better rate of response in patients treated with cetuximab **A**. The expression of β-catenin and FOXM1 was analyzed by immunohistochemistry (IHC) in 25 human colorectal cancer tumors. Representative images of IHC and hematoxylin-eosin (H-E) stainings of tumors are shown. Original magnification: X20. **B**. The association between the nuclear localization of FOXM1 and response to treatment was assessed by a chi-square test. *p* values were considered significant if they were < 0.05.

To this end, the expression of both proteins was analyzed by immunohistochemistry in a cohort of 25 advanced primary colon tumors from patients later treated with cetuximab and with distinct responses to treatment (Figure 9). A group of patients with a time to treatment failure (TTF) shorter than 6 months was defined as the non-responder group, whereas those with TTF longer than 6 months were defined as responder group. The clinicopathological features of the tumor samples analyzed are summarized in Table 1. None of the clinicopathological variables was significantly different when comparing responders and non-responders. FOXM1 and b-catenin were detected both in the cytoplasm and the nucleus of tumor cells. No significant association of the presence of b-catenin in the nucleus of tumor cells and drug response was observed. Notably, a differential subcellular localization of FOXM1 in both groups of patients was observed. Thus, as shown in Figure 8A and 8B, in most tumors (64%) from patients who responded to cetuximab therapy, FOXM1 was inmunodetected in the nucleus of tumor cells. However, this transcription factor was not immunodetected in the nucleus of tumor cells in the great majority (91%) of tumors from non-responders patients. Significantly, there was no such association between FOXM1 nuclear localization and response to treatment in samples from colorectal cancer patients treated with the anti-angiogenic drug bevacizumab, (Supplementary Figure 3). Therefore, there is a significant association between the presence in the nucleus of tumor cells of FOXM1 transcription factor and patient response to anti-EGFR treatment with cetuximab.

**Table 1:** Clinicopathological features of human colon tumors analyzed

Clinicopathological features	Responders n (%)	Non responders n (%)	*P* value^a^
**Histology**	**14**	**11**	0,1729
Adenocarcinoma	13 (93)	8 (73)	
Mucinous adenocarcinoma	1 (7)	3 (27)	
**Tumor size**	**14**	**10**	0,2850
T1	0	0	
T2	0	0	
T3	10 (71)	5 (50,0)	
T4	4 (29)	5 (50,0)	
**Nodal Status**	**14**	**10**	0,5529
N0	5 (36)	2 (20)	
N1	2 (14)	3 (30)	
N≥ 2	7 (50)	5 (50)	
**Metastasis**	**14**	**10**	0,1515
M0	12 (86)	6 (60,0)	
M1	2 (14)	4 (40,0)	
**Stage**	**14**	**10**	0,2138
I	0	0	
II	5 (36)	1 (10)	
III	7 (50)	5 (50)	
IV	2 (14)	4 (40)	

^a^None of the clinical-pathological variables were significantly different when comparing responders and non-responders to treatment with cetuximab (* p <0.05; Chi-square test).

Number of cases (n), and corresponding percentages are indicated in each case.

## DISCUSSION

Data from this study show that the combination of cetuximab and celecoxib inhibited cell proliferation, induced apoptosis and altered cell signaling pathways regulated by EGFR in K-RAS non-mutated Caco-2 and HT-29 cells. Significantly, the *in vivo* antitumor effect of the two drugs in combination was greater than each one separately. Furthermore, only the combination of both drugs reduced cell proliferation in cetuximab-resistant HT-29 cells. Interestingly, a recent study identified COX-2 as the gene with greatest difference in expression level between cetuximab-resistant and cetuximab-sensitive CRC cells [17]. On the other hand, the synergy between cetuximab and celecoxib on tumor growth inhibition, induction of apoptosis, inhibition of EGFR activity and drug resistance have been described in head and neck cancer and other tumor types[18, 19].

Studies have demonstrated that simultaneous inhibition of FOXM1 and COX-2 promotes inhibition of cell viability, and induces apoptosis in both *in vivo* and *in vitro* models of CRC [20]. In this study, we show that the combined cetuximab/celecoxib treatment of Caco-2 and HT-29 cells reduced the expression levels of FOXM1 and β-catenin and also the interaction of both proteins, particularly in the nuclear fraction. This reduction was not observed in K-RAS mutated HCT-116 cells, confirming the role of EGFR/RAS signaling in FOXM1 activation [14, 21]. Furthermore, the specific knockdown of FOXM1 in non-mutated KRAS cells significantly attenuated the anti-proliferative activity of cetuximab/celecoxib treatment, indicating that a functioning EGFR/RAS/FOXM1 signaling axis is necessary for a response to this combined treatment. Our study also shows that the combined cetuximab/celecoxib treatment significantly reduced the ability of CRC cells to form colonospheres. In agreement with these results, other study reported that simultaneous inhibition of COX-2 and FOXM1, reduced the formation of colonospheres in CRC cells [20]. Our data support that this reduction in CSC subpopulation may also be related with the regulatory role of FOXM1 on β-catenin subcellular localization, since cetuximab/celecoxib treatment significantly reduced the nuclear levels of both proteins.

Our preclinical data supports therefore a benefit by combining cetuximab treatment with celecoxib in patients with metastatic CRC. A clinical phase II trial have explored the clinical and biological effects of dual blockade of cetuximab and celecoxib in patients with metastatic colorectal cancer refractory to chemotherapy [22]. However, this clinical trial was halted early due to insufficient clinical activity since no differences in serum levels of EGFR ligand TGF-α or urinary PGE-M (stable metabolite of PGE2) between cycles were obtained, suggesting that the appropriate targets may not have been hit. It is essential to identify biomarkers that can predict response to avoid a treatment that would not benefit the patient, with consequent effects of toxicity without therapeutic value [23]. There are several studies relating a high expression of FOXM1 in mCRC with a good response to adjuvant therapy after curative surgery [24]. Other studies describe a high expression of FOXM1 in samples from mCRC patients in poorly differentiated and proliferative tumors, but with a high rate of response to treatment and propose FOXM1 as a predictive marker of response to therapy in a subset of CRC patients [25, 26]. Therefore, and given that our silencing experiments indicated that expression of FOXM1 was expected to be associated with a better response to cetuximab, in the presence or absence of celecoxib, we decided to explore whether the expression of this transcription factor could in the primary tumor be a predictor of response to treatment with this monoclonal antibody. Notably, our study shows a significant association between the nuclear localization of FOXM1 in the tumor and the response to cetuximab therapy. Besides, these data are in agreement with our *in vitro* studies showing the participation of the EGFR-RAS-FOXM1-β-catenin signaling axis in the response to cetuximab, alone or in combination with celecoxib. Therefore, the expression of FOXM1 in the tumor could be a potential predictive marker of response to cetuximab in CRC patients.

In summary, our study shows that the combination of cetuximab with celecoxib enhances the anti-tumor efficacy of both drugs in CRC cells, and reduces CSC subpopulation, due to impairment of EGFR-RAS-FOXM1-β-catenin signaling axis. Also, the present study suggests that the expression of transcription factor FOXM1 in nuclei of tumor cells could be a potential predictive marker of response of mCRC patients to cetuximab therapy.

## MATERIALS AND METHODS

### Cell culture

HT-29 and HCT-116 cells (DSMZ, Braunschweig, Germany) were grown in McCoy's 5A medium (Biowest, Nuaillé, France) containing 10% fetal bovine serum (PAA Laboratories, Pasching, Austria). Caco-2 cells (ECACC, Salisbury, UK) were grown in MEM with Earle's salts (PAA Laboratories GmbH) containing 15% fetal bovine serum. DLD-1 cells (ATCC, US ) were grown in D-MEM high glucose medium (Capricorn scientific, GmbH), containing 15% fetal bovine serum. Culture media were supplemented with 2 mM glutamine, 1% non-essential amino acids, penicillin (100 U/ml), streptomycin (100 μg/ml) and amphotericin B (2.5 μg/ml) and cells were cultured in a humidified atmosphere at 37°C and 5% CO_2_. in 96-well plates, 6-well culture plates, 60-mm culture plates or ultra low-attachment plates (Corning Inc, Lowell, MA, USA). All experiments were carried out in duplicate and repeated at least three times.

### Reagents

Cetuximab (Erbitux, Merck) was obtained from Hospital Pharmacy (5mg/ml) and stored at −20°C until use. Celecoxib (Sigma-Aldrich, Madrid, Spain) was dissolved in DMSO to produce a 20 mM stock, and stored at -20 °C. Working solutions were prepared by diluting thawed stocks into cell culture medium and diluted DMSO vehicle was used as control. Human EGF was purchased from Santa Cruz Biotechnology (Santa Cruz, CA, USA) and dissolved in glycerol to produce stock concentrations of 10% v/v. For western blot analysis, the following antibodies were used: phospho-EGF Receptor (Tyr1068) rabbit pAb, phospho-p44/42 MAPK (Erk 1/2) (Thr202/Tyr204) (D13.14E) rabbit mAb, phospho-Akt (Thr308)(C31E5E) rabbit mAb, phospho-Akt (Ser473)(587F11) mouse mAb, Akt rabbit pAb and β-Catenin rabbit pAb were purchased from Cell Signaling Technologies (Danvers, MA, USA). EGFR mouse mAb (0.N.268), actin (C-2) mouse mAb, goat anti-rabbit, goat anti-mouse, donkey anti-goat secondary antibodies, and FOXM1 (K-19): sc-500 were purchased from Santa Cruz Biotechnology. MAP kinase ERK1/ERK2 rabbit pAb was from Calbiochem-EMD Millipore (Billerica, MA, USA). Tubulin (DM1A) mouse mAb (ab80779), was purchased from abcam (Biotech, Life sciences). Propidium iodide was obtained from Sigma-Aldrich and was prepared by dissolving 1 mg in 1 ml phosphate buffered saline. This solution was protected from light and stored at 4 °C. RNase, DNase-free was obtained from Roche Applied Science (Indianapolis, IN, USA). Stock concentrations of 500 μg/mL RNase were prepared and kept at – 20°C.

### Cellular proliferation assay

Cell proliferation was assayed using an IncuCyte® ZOOM Live-Cell Analysis System (Essen BiosScience, Ann Arbor, Michigan), by collecting real-time data of cell confluence. Cells were seeded on 96-well plates (4,000 cells/well) and let to attach for 24 hours. The cells were then treated with cetuximab (100 mg/ml) as a single agent or in combination with celecoxib (10 μM or 50 μM). Control cells were treated with the same concentration of the DMSO vehicle. Cell proliferation data were obtained by the cell confluence increment in each of the treatments and expressed as percentage relative to that of control cells.

### Colonosphere formation assay

Treated cells were tripsinized and re-seeded in ultra-low attachment 96-well plates (Costar, Corning, NY, USA) at clonal density (1 cell/μl) in serum free Dulbecco's MEM Nutrient Mixture F+12 Ham medium supplemented with B27 (1:50; Invitrogen, Carlsbad, CA, USA), 10 ng/ml basic fibroblast growth factor (PeproTech, London, UK)), 20 ng/ml EGF (Santa Cruz Biotechnology) and 1% v/v methylcellulose (R&D Systems, Minneapolis, MN, USA) to prevent cell aggregation. The supplements were freshly added every 2-3 days and the number and size of formed colonospheres were evaluated on day 7 after seeding using the IncuCyte® ZOOM Live-Cell Analysis System.

### Western blotting analysis

After treatments cells were harvested with cold PBS and centrifuged at 300 x *g*, for 5 minutes at 4°C. Pelleted cells were lysed for 15 minutes on ice with 1 ml lysis buffer (50 mM Tris-HCl (pH 7.4), 150 mM NaCl, 5 mM ethylenediamine tetraacetic acid (EDTA), 1 mM ethyleneglycol tetraacetic acid (EGTA), 1.5 mM MgCl_2_, 10% glycerol, 1% NP40, 0.1 M dithiothreitol (DTT), 0.1 M phenylmethylsulfonyl fluoride (PMSF), 1% v/v protease inhibitor cocktail (SERVA, Heidelberg, Germany) and 1% v/v phosphatase inhibitor cocktails 2 and 3 (Sigma-Aldrich) and centrifuged at 10,000 x *g* for 15 minutes at 4°C. Protein concentration was quantified by a standard Bradford assay using the colorimetric reagent from BioRad Laboratories (Hercules, CA, USA). Proteins (12.5 μg) were separated onto SDS polyacrylamide gels using a 4-12% Bis-Tris gradient gels in the BioRad Criterion System, transferred to nitrocellulose membranes. After blocking with 3% BSA, membranes were incubated with the specific antibodies. Inmunocomplexes were detected with appropriate horseradish peroxidase-conjugated secondary antibodies and enhanced chemiluminescence (ECL Plus Western Blotting Detection System or ECL Advance Western Blotting Detection Kit, GE Healthcare Life Sciences, Little Chal-font, UK). Images were digitalized on a ChemiDoc XRS Imaging System (BioRad Hercules, CA, USA).

### Combined annexin-V/propidium iodide staining

After 72h of treatment of cells with cetuximab and/or celecoxib (6-well plates, 3 × 10^6^ cells/well) the fraction of apoptotic cells was estimated by using an Annexin-V/propidium iodide (PI) staining kit (Bender MedSystems, Viena, Austria), according to manufacturer´s recommendations. Binding of fluorescein-conjugated Annexin-V and PI was measured by flow cytomewtry (FACSCalibur; BD, Franklin Lakes, NJ, USA) to quantify the percentage of apoptotic cells.

### Cell cycle analysis

Cells (0.5 -1×10^6^ cells) were trypsinized and resuspended in PBS. Then, ice-cold 100% ethanol was added in a drop-wise manner while gently vortexing and incubated for 20 minutes at room temperature. Samples were centrifuged at 300×*g* for 5 minutes, resuspended in PBS containing 50 μg/ml propidium iodide plus 100 μg/ml RNase A and incubated for 20 minutes at room temperature protected from light. The analysis and measurement of propidium iodide fluorescence were performed on a FACSCalibur (BD Biosciences) flow cytometer (FACS; BD, Franklin Lakes, NJ, USA).

### Transient expression of mutant K-RAS gene in Caco-2 and HT-29 cells

Caco-2 and HT-29 cells were transiently transfected with pBabe K-Ras 12V plasmid or the empty vector (pBABE-puro), provided by Addgene (plasmids #12544 and #1764). PureYield Plasmid Midi-prep System (Promega) was used following the protocol supplied by the manufacturer. Cells were seeded at 90% confluence in 6‐well plates and after 24 h cells were transfected using Lipofectamine 3000 (Life Technologies) as transfection reagent, following the manufacturer's instructions.

### Mouse xenograft model and treatments

Caco-2 cells (3 × 10^6^) in 200 μl of matrigel (BD Biosciences) were subcutaneously injected into the both flanks of 5-weeks old male NOD-SCID mice (NOD.CB17-Prkdc scid/NcrCrl) (Janvier Labsa, Le Genest-Saint-Isle, France). When tumors reached approximately 100–150 mm^3^ volume (2 weeks after of inoculation) mice were randomly assigned into four groups (each group included 3 mice, six tumors). Treatments were intraperitoneally administered on days 1 and 5 of each week for 4 weeks. Control group received vehicles (DMSO and saline), the CXB group was treated with 60 mg/kg celecoxib, the CTX group was treated with 20 mg/kg cetuximab and the CXB + CTX group received 60 mg/kg celecoxib plus 20 mg/kg cetuximab. The length and width of each tumor were measured with a digital caliper and tumor apparent volumes were calculated [V (mm^3^)= (Length x [width]^2^ )/2.

### Knockdown of FOXM1 with specific small interfering RNA

FOXM1 gene specific small interfering RNA [L-009762-00-0005, ON TARGETplus Human FOXM1 (2305) siRNA-SMARTpool] and non-specific siRNA were obtained from GE Dharmacon (Lafayette, CO, USA) and used according to manufacturer´s instructions. In brief, siRNA was dissolved (10 nM) in Optimen (1x-Reduced Serum Medium, Gibco) and incubated with lipofectamine 2000 (Thermo Fisher Scientific) for 10 min at room temperature. Cells were incubated with the transfection complexes under normal growth conditions for 24 h, then the medium was replaced to perform the different treatments in the transfected cells.

### Immunofluorescence confocal microscopy

Cells were grown on poly-L-Lysine-treated coverslips and after 6h of the different treatments the culture media was discarded, cells were washed thrice in PBS and permeabilized in methanol. After washing with PBS, coverslips were incubated with Anti-b-Catenin (1/250) mouse mAb (BD) and FOXM1 (1/250) rabbit pAb (Santa Cruz Biotechnology), for 1h at room temperature. Coverslips where washed thrice in PBS and incubated for 1h at room temperature with an anti-mouse IgG alexa fluor 488-labeled antibody (1/500) (Molecular Probes, Eugene, OR, USA) and with anti-rabbit IgG (H+L) alexa fluor 594-labeled antibody (1/500) (Santa Cruz Biotechnology). Finally, cells were incubated with 300 nM 4′,6-diamidino-2-phenylindole dihydrochloride (DAPI) in PBS for 5 minutes at room temperature. Coverslips were mounted using Aqua Poly/Mount (Polysciences, Warrington, PA, USA) and visualized in a Zeis LSM 5 Exciter Confocal laser-scanning microscope (Zeiss, Germany). Images were analyzed using the image-J software (National Institutes of Health).

### Immunohistochemical analyses

Immunohistochemical analyses were performed on human adenocarcinoma paraffin-embedded samples obtained from the biobank of Hospital Reina at Córdoba. These samples were from tumors of CRC patients treated with cetuximab. Tumors of CRC patients treated with bevacizumab (Avastin) were used as controls. The signed informed consent of patients for participation in this study and approval of the ethics committee board of the University Hospital Reina Sofía of Córdoba was previously obtained. Paraffin sections (3 μm) on poly-L-lysine-coated slides were used after drying for 30 min at 60°C. The sections were dewaxed in xylene, rehydrated in ethanol and incubated at 100°C in citrate buffer pH 9,0 (EnVisionTM Flex DM812-Dako) for 5 min for antigen retrieval. After washing in PBS, the sections were incubated for 10 min in 3% hydrogen peroxide to block endogenous peroxidase, and then incubated monoclonal anti-FOXM1 antibody (HPA029975, Sigma-Aldrich) overnight at 4°C. After washing 5 min in PBS, the slides were incubated 30 min with EnVision FLEX+rabbit (Dako) to amplify signal and then incubated 30 min with a HRP-labelled polymer (EnVisionTM Flex/HRP SM802-Dako) and developed for 3 min using diaminobenzidine. Finally, the slides were counterstained with hematoxylin and mounted in Eukitt mounting medium. Microscopy images were obtained using a Coolscope digital microscope (Nikon, Tokio, Japan). Sections were examined scored by two experienced pathologists.

### Statistical analysis

All data are expressed as mean ± standard error of mean. All Statistical analyses were performed using GraphPad Prism 5. Before comparing two data groups, a normality test and an equal variance test were performed. If data groups passed both tests, a comparison was made by a parametric approach (paired Student's t-test). If the normality and/or equal variance test was violated, a comparison was made by a nonparametric method (Mann-Whitney test). The differences between responders and non-responders patients to therapy with cetuximab and bevacizumab were evaluated by chi-square test. Differences were considered statistically significant at p < 0.05.

## SUPPLEMENTARY MATERIALS FIGURES


