# Supplementary material for: The addition of celecoxib improves the antitumor effect of cetuximab in colorectal cancer: role of EGFR-RAS-FOXM1-β-catenin signaling axis
Source: Oncotarget. 2017 Feb 21;8(13):21754–69. doi: 10.18632/oncotarget.15567 (PMC5400621; doi:10.18632/oncotarget.15567)
Supplement: Supplementary file 1 [file oncotarget-08-21754-s001.pdf]

The addition of celecoxib improves the antitumor effect of cetuximab in colorectal cancer: role of EGFR-RAS-FOXM1-β-catenin signaling axis

SUPPLEMENTARY FIGURES

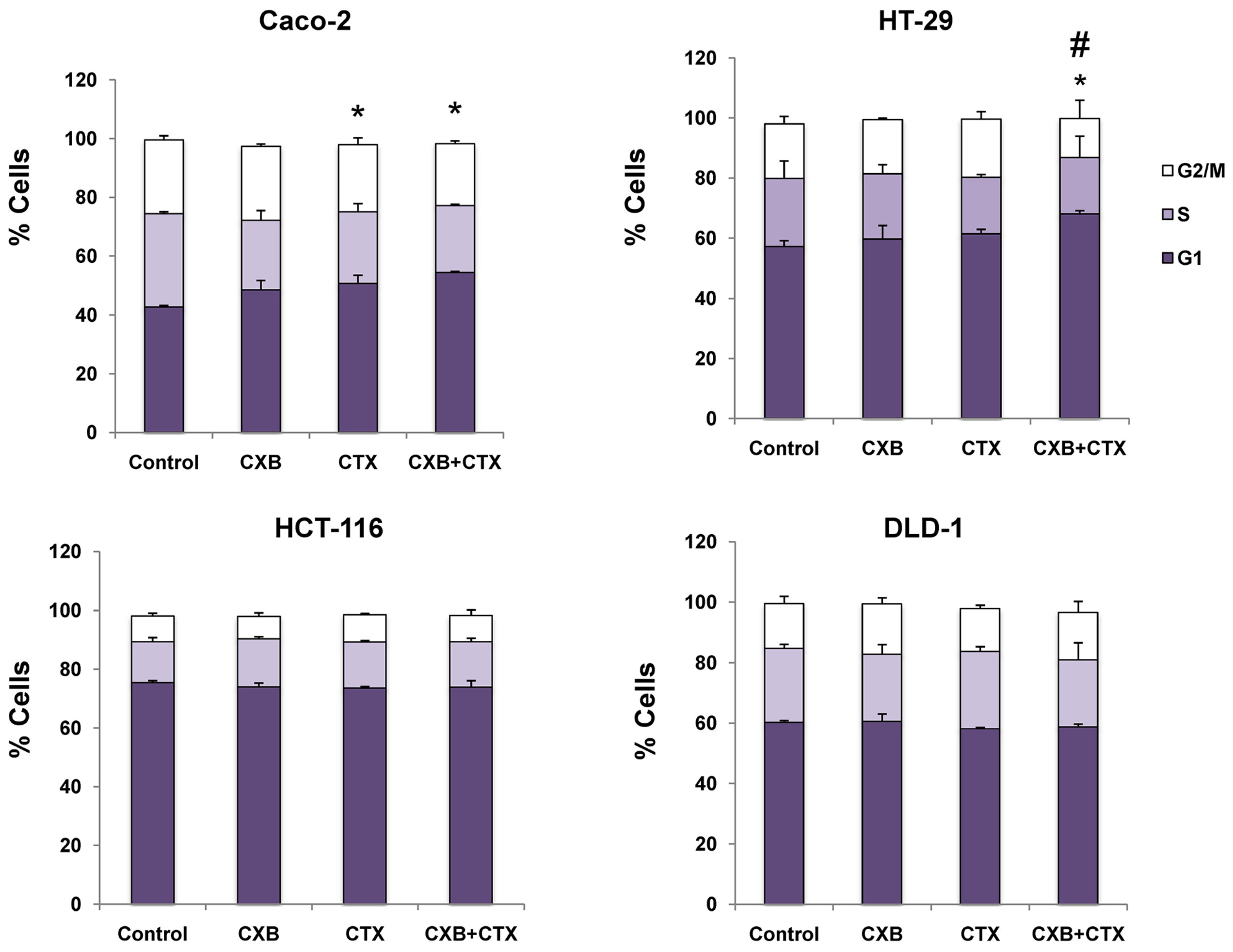

Supplementary Figure 1: The combination of cetuximab with celecoxib alters cell cycle in colorectal cancer cells. Cell cycle analysis was performed by flow cytometry after 48 h of treatment with cetuximab (CTX, 100 µg/ml) and/or celecoxib (CXB, 50 µM) of cells growing in the presence of EGF (100 ng/ml). Data are means ± SEM of three independent experiments (*p <0.05, compared with the control; # p<0.05, compared with cetuximab-treated cells).

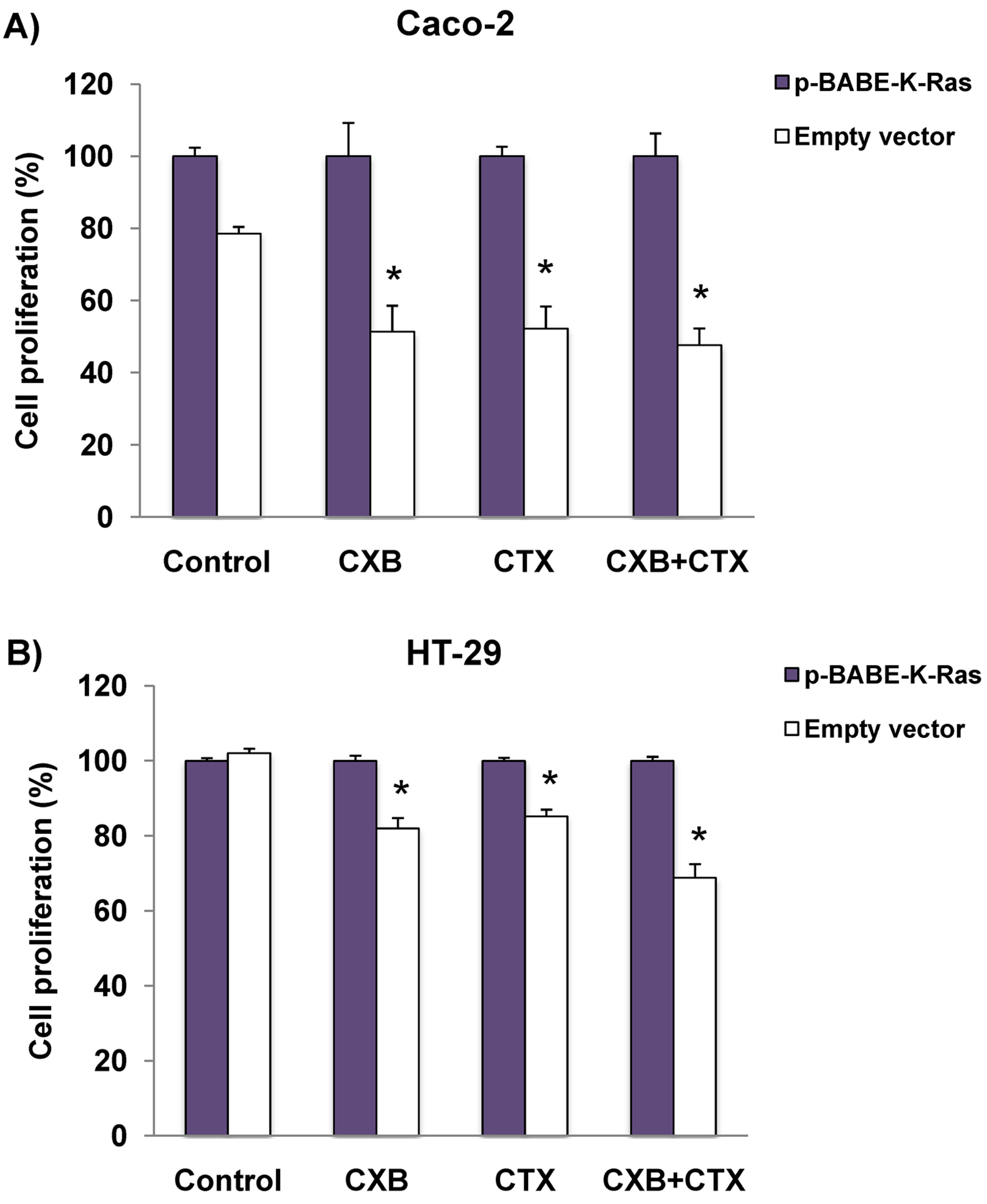

Supplementary Figure 2: Transient expression of mutant K-RAS gene in Caco-2 and HT-29 cells reduces the antiproliferative effects of the combination of cetuximab with celecoxib. **A.** Caco-2 and **B.** HT-29 cells transiently transfected with pBabe K-Ras 12V plasmid showed reduced sensitivity to the antiproliferative effect of cetuximab and celecoxib. Data are means ± SEM of three independent experiments (*p <0.05, compared with the mutant K-RAS transfected cells).

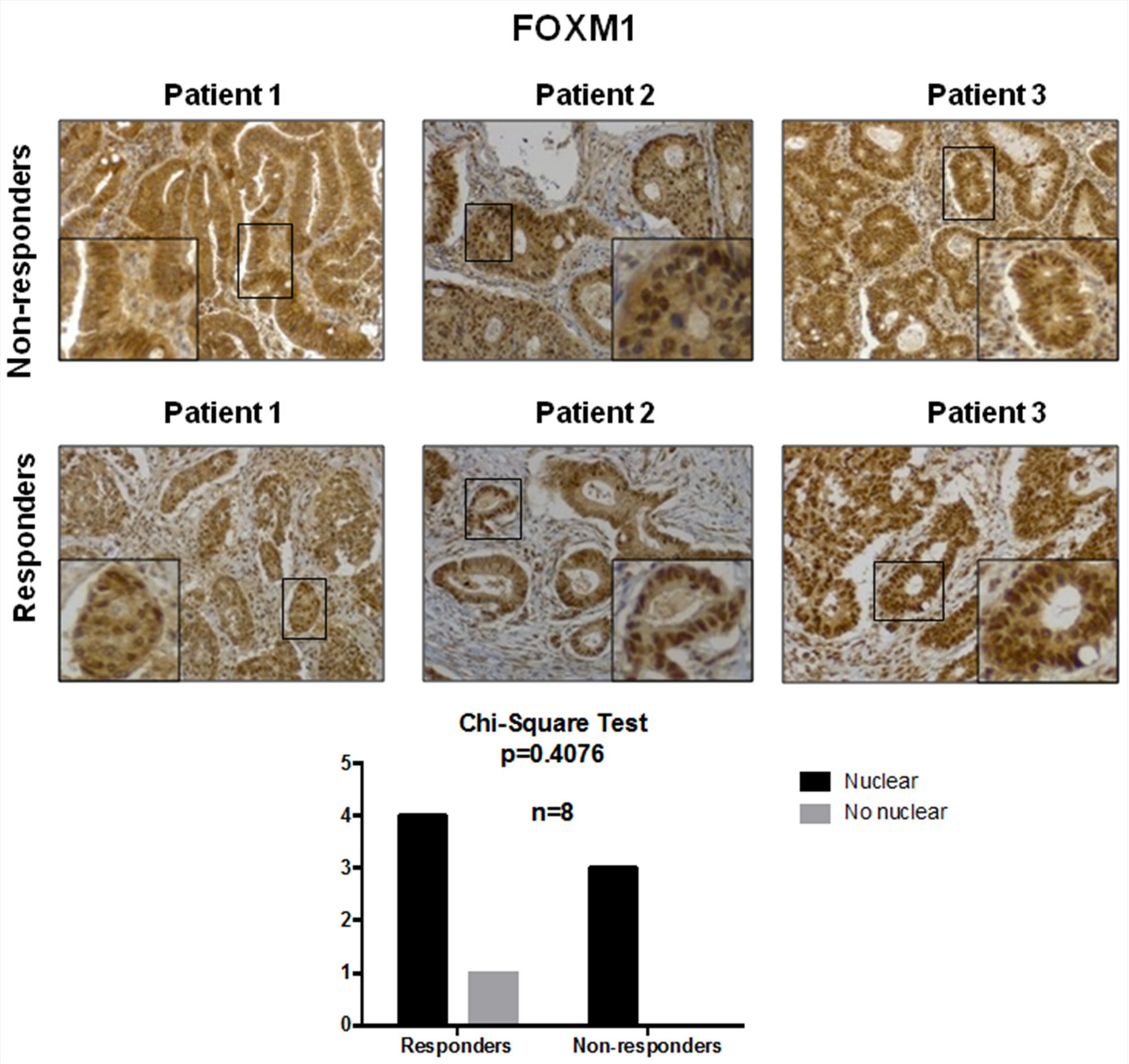

Supplementary Figure 3: FOXM1 expression in colon cancer and response of patients to the anti-angiogenic drug bevacizumab. The expression of FOXM1 was analyzed by immunohistochemistry (IHC) in 8 human colorectal cancer tumors. **A.** Representative images of IHC stainings of tumors are shown. **B.** The association between the nuclear localization of FOXM1 and response to treatment was assessed by a chi-square test and p values were considered significant if they were < 0.05.
